# Supplementary material for: A Humanized Yeast Phenomic Model of Deoxycytidine Kinase to Predict Genetic Buffering of Nucleoside Analog Cytotoxicity
Source: Genes (Basel). 2019 Sep 30;10(10):770. doi: 10.3390/genes10100770 (PMC6826991; doi:10.3390/genes10100770)
Supplement: Supplementary file 1 [file genes-10-00770-s001.zip › Additional_Files/Additional_File7_GOTermHeatmaps.zip!/Additional_File7_GOTermHeatmaps/ribophagy.pdf]

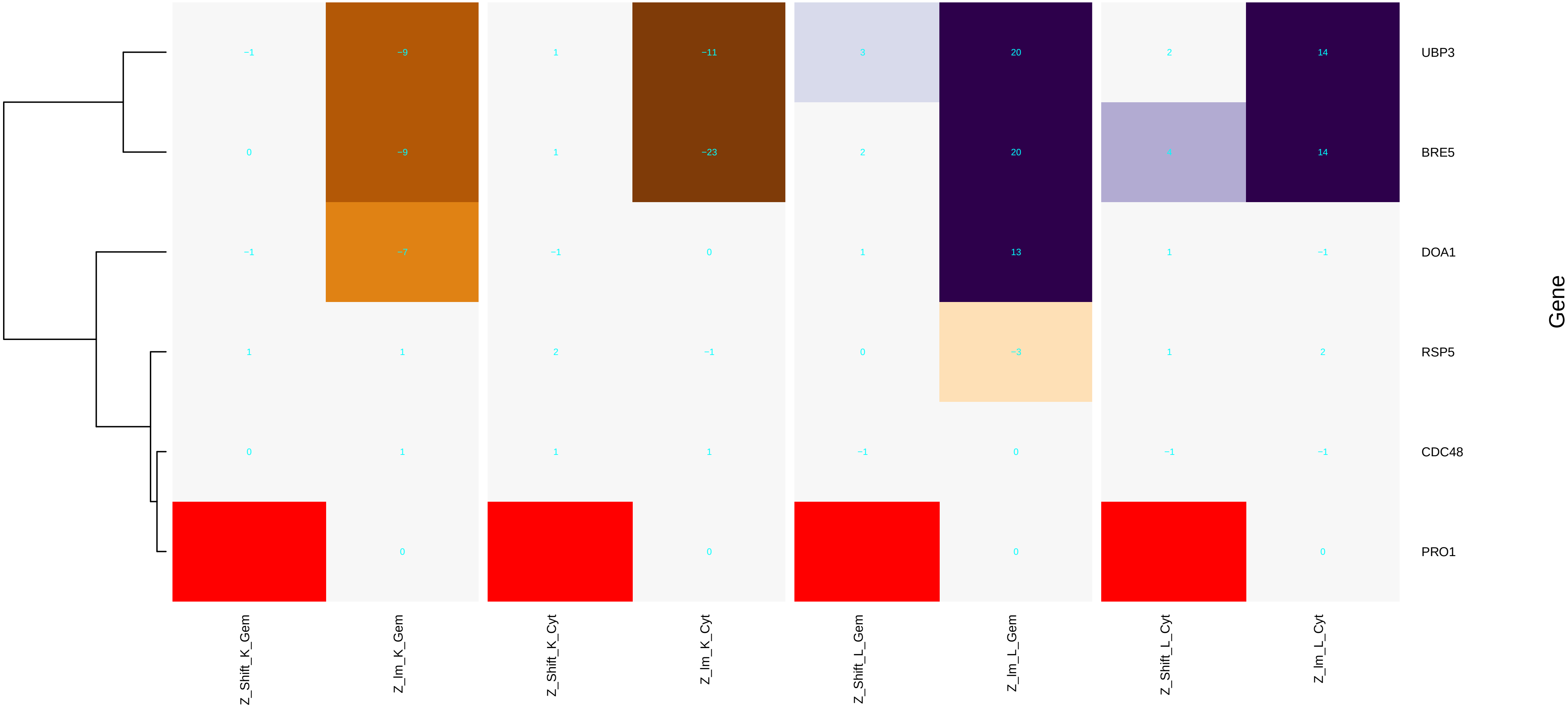

UBP3
BRE5
DOA1
RSP5
CDC48
PRO1
Gene
Z_Shift_K_Gem
Z_Im_K_Gem
Z_Shift_K_Cyt
Z_Im_K_Cyt
Z_Shift_L_Gem
Z_Im_L_Gem
Z_Shift_L_Cyt
Z_Im_L_Cyt
−1 −9 1 −11 3 20 2 14
0 −9 1 −23 2 20 4 14
−1 −7 −1 0 1 13 1 −1
1 1 2 −1 0 −3 1 2
0 1 1 1 −1 0 −1 −1
0 0 0 0
